# Expression pattern of the apoptosis-stimulating protein of p53 family in p53^+^ human breast cancer cell lines

**DOI:** 10.1186/1475-2867-13-116

**Published:** 2013-11-18

**Authors:** Changsong Wang, Chunfang Gao, Yanping Chen, Jian Yin, Ping Wang, Xuexia Lv

**Affiliations:** 1 Department of Pathology, 150th Hospital of PLA, Luoyang, Henan Province, China

**Keywords:** ASPP family member, Breast cancer cell line, Apoptosis, p53

## Abstract

**Background:**

The apoptosis-stimulating protein of p53 (ASPP) family comprises three members, namely, ASPP1, ASPP2, and iASPP. They regulate the promotive effect of p53 on apoptosis. Breast cancer (BC) remains as one of the leading causes of cancer or cancer-related mortality among women. However, the relationship between the ASPP family members and p53, as well as the dissemination and expression pattern of ASPP family members in p53^+^ BC, has not been elucidated. Our objectives are to detect the expression of ASPP family members in p53^+^ BC cell lines and determine its significance in tumor cell apoptosis.

**Methods:**

The mRNA expression of ASPP family members in five p53^+^ BC cell lines was detected through RT-PCR and assayed using Quality-one software. The p53 protein expression was detected by immunohistochemistry. Afterward, the apoptosis indices of the five BC cell lines were detected by flow cytometry.

**Results:**

The iASPP mRNA was expressed in Bcap-37, MCF-7, and HBL-100. Compared with the human peripheral blood mononuclear cells, significant differences were found in the ASPP1 mRNA in Bcap-37, MDA-MB-231, MCF-7, and HBL-100 (*p* < 0.05), except that in ZR-75-30 (*p* > 0.05). The ASPP2 mRNA was expressed in MDA-MB-231, Bcap-37, and MCF-7, but not in HBL-100 and ZR-75-30. The p53 protein was expressed in five breast cancer cell lines. ZR-75-30 and MDA-MB-231 apoptosis indices were higher than those of other breast cancer cell line and peripheral blood mononuclear cells (*p* < 0.01).

**Conclusions:**

The mRNA expression of ASPP family members varied in the five p53^+^ BC cell lines. The results also verified that the family members have an important function in apoptosis, which was promoted by p53 protein. ZR-75-30 BC showed high apoptosis index, without expression of any ASPP family members, indicating that the pathway of apoptosis in this cell line may be related to other cell transduction pathway. MDA-MB-231, Bcap37, and MCF-7 cell lines all expressed ASPP1/2. However, the apoptosis pathway in MDA-MB-231 is different from those of the other two cell lines. The status of the different cell lines should also be considered when the functions of the ASPP family members are examined.

## Background

Breast cancer (BC) is the most common cancer among women worldwide [1-4], with 692,200 and 691,300 patients in developed and developing countries, respectively, in 2008 [5]. During BC development, genetic and non-genetic factors have key functions such as in the overexpression and/or underexpression, polymorphism, mutation and/or deletion of a few specific genes or group of genes. More molecules have been found to support the individual treatment and molecular classification of BC. The apoptosis-stimulating protein of p53 (ASPP) family has recently been reported as an apoptotic specific regulator of p53 [6-9]. ASPP family members include three members: iASPP, which is an inhibitory member that binds with p53 and interferes with its apoptosis-induction ability [8]; and ASPP1 and ASPP2 that bind with p53 protein to induce apoptosis of DNA-damaged cells. ASPP family members are important factors in the apoptosis pathway, which is triggered by p53 protein. A few published reports have shown that the function of p53 protein is regulated by inhibiting iASPP over-expression in cancer cells to remove its interference on p53 when iASPP cancer cells are overex-pressed [10,11]. Elucidating the expression of ASPP family members in BC cells, along with a better understanding of the relationship between the expression of individual ASPP family members and BC cell line apoptosis, may facilitate the development of effective strategies that provide effective therapy protocols to BC patients. Investigating the functions of the ASPP family members in in vitro expression in human BC cell lines, such as in the ER-negative MDA-MB-231 (highly invasive and metastatic cells) and ER-positive MCF-7 (weakly invasive and metastatic cells), offers an approach to better understand the relation of the ASPP family in BC evolution. Majority of the available data on the expression of the ASPP family members in BC cell lines are somehow contradicting. Hence, examining the expression of these members in different human BC cell lines is important.

## Results

### Evaluation of the mRNA expression of ASPP family members by reverse transcription-polymerase chain reaction (RT-PCR)

**Figure 1:**
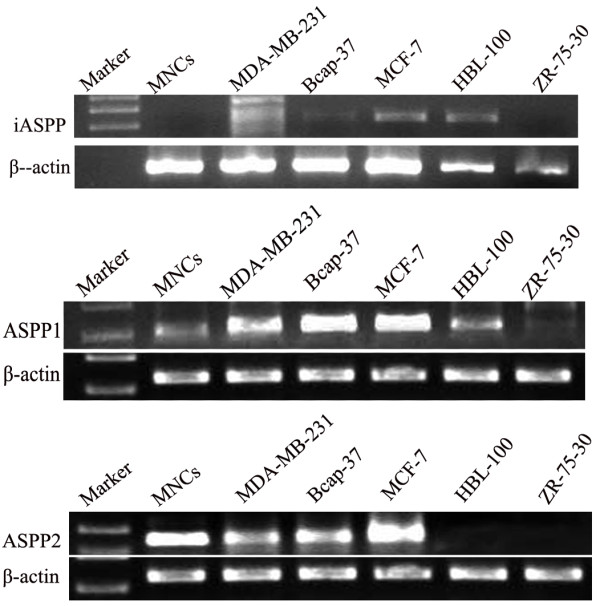
Expression of ASPP family members mRNA in 5 BC cell lines.

ASPP1 mRNA expression was observed in four BC cell lines (i.e., MDA-MB-231, Bcap-37, MCF-7, and HBL-100 cells). The expression levels were higher in MDA-MB-231, Bcap-37, and MCF-7 than that in the mononuclear cells (MNCs) and HBL-100. In addition, no expression was observed in ZR-75-30. Higher ASPP2 mRNA expression was observed in MNCs, MDA-MB-231, Bcap-37, and MCF-7, but no ASSP2 expression was observed in HBL-100 and ZR-75-30. iASPP mRNA expression was observed in MCF-7 and HBL-100, slight expression was observed in Bcap-37, and no expression was found in MNCs, MDA-MB-231, and ZR-75-30 (Figure 1).

### Analysis of the expression of the ASPP family members using Quality-one image scan

The optical density value of the ASPP family members and β-actin were analyzed using Quality-one image scan software, and the results showed the quantity of the relative expression in the five BC cell lines. iASPP expression was observed only in Bcap-37, MCF-7, and HBL-100. ASPP1 mRNA expression was significantly different among Bcap-37, MCF-7, HBL-100, MDA-MB-231, and MNCs (p < 0.05), except in ZR-75-30. ASPP2 mRNA levels in MDA-MB-231, Bcap-37, and MCF-7 cell lines were higher than that in MNCs (p < 0.05). No ASPP2 expression was detected in HBL-100 and ZR-75-30 (Table 1).

**Table 1:** The ratio of the integrated optical density (IOD) of ASPP family members and β-actin in BC cell lines

	**MNCs**	**MDA-MB-231**	**Bcap-37**	**MCF-7**	**HBL-100**	**ZR-75-30**
iASPP	0	0	0.3236 ± 0.0129	0.7052 ± 0.0229	0.7212 ± 0.0191	0
ASPP1	0.7612 ± 0.0143	1.0233 ± 0.0114^*^	1.0495 ± 0.0220^*^	1.0209 ± 0.0112^*^	0.9925 ± 0.0038^*^	0
ASPP2	0.6180 ± 0.0036	0.9981 ± 0.0457^*^	1.0576 ± 0.0194^*^	1.1667 ± 0.0119^*^	0	0

vs MNCs, *P < 0.05.

### P53 protein expression by immunochemistry and Western blot analysis

**Figure 2:**
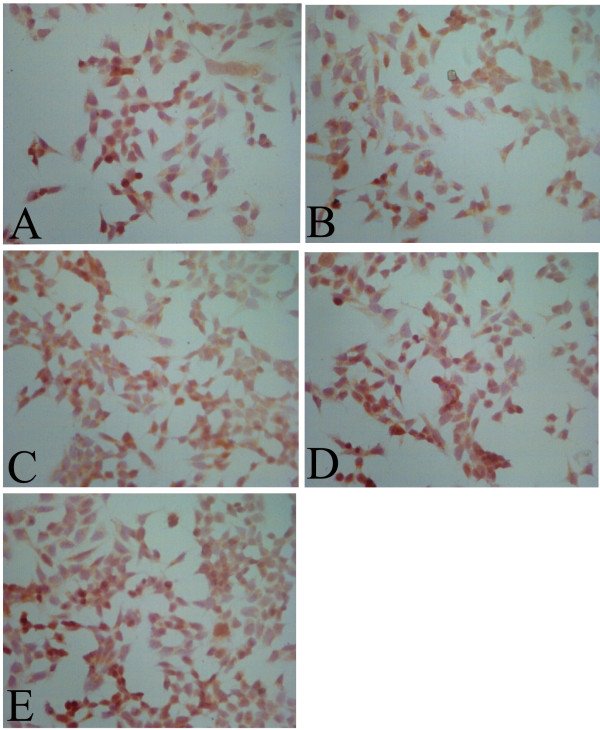
**Expression of p53 in both BC cell lines. (A)** MDA-MB-231. **(B)** ZR-75-30. **(C)** Bcap-37. **(D)** MCF-7. **(E)** HBL-100 (×200 magnification).

**Figure 3:**
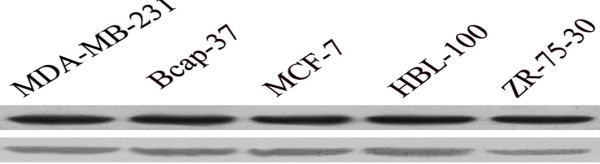
Western-blot result of p53 protein expression in 5 BC cell lines.

The positive cells showed yellow nuclei. All five BC cell lines reacted positively (Figure 2). Thus, p53 protein was expressed in the five BC cell lines (Figure 3).

### Apoptosis detection

To further explain the relationship between ASPP family members and BC cells, the five BC cell lines were cultured in vitro. Cell apoptosis was determined using flow cytometry (FCM). The results are shown in Table 2. Apoptosis was found to be different among the cell lines without any interference. The apoptosis indices of ZR-75-30 and MDA-MB-231 cell lines were higher than those of Bcap-37, MCF-7, HBL-100, and MNCs (p < 0.01).

**Table 2:** **Apoptosis index of BC cell lines (%,**$\bar{x}$**±s)**

**Cell lines**	**Apoptosis index**
MDA-MB-231	8.4 ± 0.18^*^
ZR75-30	8.9 ± 0.23^*^
Bcap-37	3.1 ± 0.73
MCF-7	2.1 ± 0.12
HBL-100	3.6 ± 1.04
MNCs	2.3 ± 0.13

*vs Bcap-37、MCF-7、 HBL-100 and MNCs, (P < 0.01).

### Apoptotic changes in ZR-75-30 and MDA-MB-231 through transfection with pAd-p53-RNAi

To further demonstrate the relationship between p53 protein and the BC cell lines, we transfected the BC cell lines ZR-75-30 and MDA-MB-231, with high apoptosis indices, using pAd-p53-RNAi. Cell apoptosis was determined via FCM post-pAd-p53-RNAi transfection. The results are shown in Table 3. The cell apoptosis index decreased markedly after the interference plasmid was transfected.

**Table 3:** **Apoptosis index of BC cell lines after transfected p53 RNAi (%,**$\bar{x}$**±s)**

**Cell lines**	**Apoptosis index**
MDA-MB-231	3.4 ± 0.11
ZR75-30	2.6 ± 0.37
MNCs	1.9 ± 0.44

## Discussion

The wild-type p53 is an important anti-oncogene and its expression induces tumor cell apoptosis. P53 protein binds with the DNA of damaged cells, which results in cell cycle arrest. Afterward, the damaged cells are repaired by the host or killed via apoptosis. Numerous studies have shown that p53 protein increased markedly in DNA-damaged cells. The process by which p53 protein induces cell apoptosis has not been elucidated. The recent discovery of ASPP explained the p53-induced cell apoptosis because this protein is a specific p53 regulator [6-8]. The ASPP family is also the most effective protein family that regulates p53 protein [6,12,13]. The ASSP family comprises ASSP1, ASSP2, and iASPP, which have diverse functions. ASSP1 and ASSP2 can activate p53 to induce cell apoptosis, but iASPP inhibits p53 protein function. Therefore, p53-induced cell apoptosis can be activated by ASSP1 and ASSP2 but can be inhibited by iASPP.

To date, the status and expression pattern of the ASPP family in BC cell lines have not been reported, and whether the ASPP family could be a new therapy target in BC patients remain unknown. In the current study, we analyzed the mRNA expression pattern of the ASPP family in five p53+ BC cell lines using semi-quantitative RT-PCR method. The results revealed that ASPP1 was expressed in four BC cell lines. ASPP2 was expressed in MDA-MB-231, Bcap-37, and MCF-7. iASPP was expressed only in MCF-7, HBL-100, and Bcap-37. To further study the relationship between the ASPP family members and p53+ BC cell lines, we detected the apoptosis indices of untreated BC cell lines. Results showed that the apoptosis indices of MDA-MB-231 and ZR-75-30 (non-expression iASPP) were markedly higher than those of the other BC cell lines. Our results further verified the relationship between the ASPP family members and apoptosis in p53+ BC. Apoptosis induction in p53+ BC also involves other pathways aside from the ASPP family transduction pathways.

According to a previous study, 4.1% and 1.5% of p53 binds with ASPP1 and ASSP2, respectively, in normal cultural MCF-7 BC cell line, p53-ASPP2 complexes increase to 3.4 times after the cells are UV-irradiated, and p53-ASPP1 complexes remain unchanged [6]. These results revealed that protein binding of p53 with ASPP2 increased markedly in DNA-damaged cells. Consequently, inhibition of iASPP expression in MCF-7 cells by RNA interference strategy increased apoptosis [14]. The binding ability of ASPP1 with mutant-p53 was weaker than that with wild-p53 protein. In addition, p53-DNA complexes changed when ASPP1 and ASSP2 slightly changed, consequently altering tumor cell apoptosis. In the present study, the results revealed that the expression of the ASPP family members vary among the different p53+ BC cell lines. P53 expression was also detected in the five BC cell lines, and we infer that this difference was related to the status of the p53 gene. P53 mutation was noted in approximately 30% of patients with breast cancer. Thus, 70% of patients with breast cancer exhibit wild-type p53. However, the prognosis and recurrence rate vary among patients, which may be related to the status of p53 and the ASPP family members. Therefore, we believe a new strategy must be used in treating different BC patients.

Finally, despite the increased awareness on the relationship between the ASPP family and BC, many questions remain unresolved, particularly regarding the functions of the ASPP family members in p53^+^ BC cell lines. A few reports have shown that ASPP1 and ASPP2 act as tumor suppressors and iASPP as an oncogene [15,16]. iASPP over-expression has been found in various tumor cells [6,17]. Inhibition of iASPP over-expression may also be a new strategy to continue the cancer-suppressing function of p53 protein. ZR-75-30 BC, which did not express any ASPP family member, exhibited a high apoptosis index. This finding indicated that the apoptosis pathway in this cell line may be related to other cell transduction pathway. Simultaneously, different apoptosis indices were observed in ASPP1 and ASPP2 expression BC cells (MDA-MB-231, Bcap37, and MCF-7), indicating that apoptosis pathways were diverse in these BC cell lines. These results predicted that an intermediate link existed between the ASPP family and p53. Two recent studies have reported that ASPP1 and ASPP2 bound with RAS to enhance p53 activity in cancer cells [18]. ASPP1 inhibited apoptosis by controlling YAP function [19], and the ASPP family members interacted with other p53 family members (p63 and p73) to influence apoptosis effects [20]. These intermediate links probably caused the difference in the apoptosis indices in the p53+ BC cell lines. Our results also verified that the expression pattern of the ASPP family members was altered in almost 80% of human BC cases, which was reported by Bergamaschi et al. [13]. The ASPP family members may be used as a new target for BC treatment [21,22], and the investigation of the function of the ASPP family should consider the status and characteristics of the ASPP family members in p53^+^ BC cell lines.

## Materials and methods

### BC cell lines

MDA-MB-231, Bcap-37, MCF-7, HBL-100, ZR-75-30, and MNCs were maintained in Dulbecco modified Eagle medium supplement with 100 IU/mL penicillin-G, 100 μg/mL streptomycin sulfate, (Gibco/Life Technologies, Grand Island, NY, USA), and 10% fetal bovine serum (FBS, Natocor, Argentina) at 37°C under 5% CO_2_ in a humidified atmosphere. The medium was changed every 3 d to 4 d. The cells were used up to passage 4. The experimental protocols were approved by the ethics committee of the 150th Hospital, PLA (20121008023).

### RNA preparation

Total RNAs were extracted from the five BC cell lines using Tripure isolation reagent (Roche, USA).

### RNA expression determination by RT-PCR

RT-PCR kits (TaKaRa, Japan) were used to synthesize cDNA from 5 μL of total RNA. cDNA synthesis was performed, as suggested by the kit protocol, using AMV reverse transcriptase. Reverse transcription was performed for 2 min at 94°C for one cycle, and then at 94°C for 40 s, at 72°C for 40 s for 30 cycles, and finally at 72°C for 10 min for one cycle extension. Appropriate temperatures for ASPP1, ASPP2, and iASPP were 62, 59, and 60°C, respectively. cDNA (5 μL) was used in amplifying the target regions of ASPP1, ASPP2, or iASPP. β-Actin cDNA fragments were also amplified as positive control. The RT-PCR primers of ASPP1, ASPP2, and iASPP were designed using Primer Premier 5.0 software (Table 4). The nucleotide sequences of these primers and their PCR conditions are summarized in Table 1. After PCR, 10 μL of product was mixed with 1 μL of 10× loading dye, and was subsequently placed on 1.4% agarose gel. Electrophoresis was performed at 70 V under ambient temperature. The bands on the gels were visualized using ethidium bromide staining.

**Table 4:** Primers of amplified ASPP family members

	**Sense primer**	**Anti-sense primer**	**Product size**
ASPP1	5′-CAAATGCTGCTCATGGAAGA-3′	5′-CAGGACCCAGAGGTGTAGGA-3′	300 bp
ASPP2	5′-GCCGATGTTTCTTACCGTGT-3′	5′-ATTCGCTCATTATCCGCAAC-3′	180 bp
iASPP	5′-GATGAACTGACCAAGCA-3′	5′-CTCCCTCCAAGGCAAC-3′	753 bp

### RT-PCR production analysis

The lengths of the PCR products of ASPP1, ASPP2, and iASPP were 300, 180, and 753 bp, respectively. Electrophoresis profiles were analyzed using Quality-one image scan software (Bio-Rad Inc., USA). The ratio of the integrated optical density of ASPP and β-actin represented the mRNA level.

### Immunocytochemistry

The BC cell lines were used to evaluate the p53 protein expression thrice via immunocytochemistry assays. BC cells (6 × 10^5^ cells/well) were fixed using acetone solution. Endogenous peroxidase activity was then blocked, and the BC cells were incubated with primary human Abs, 10 μL anti-p53 (AB1862, Chemicon, 1:20), and anti-AE1AE3 as a positive control (IR053, Dako). According to the manufacturer’s recommendations, a peroxidase-based immunocytochemistry staining method (K0690, Dako) was used for primary Ab detection, and a 3,3′-diaminobenzidine tetrahydrochloride substrate system (K3468, Dako) was used as chromogen. Negative controls without primary Abs were also prepared at the same time. Each sample was assayed in quadruplicate.

### Detection of p53 protein expression using Western blot analysis

Cells were collected and lysed in RIPA lysis buffer (50 mM Tris–HCl, 150 mM NaCl, 1% NP-40, 0.1% SDS, 5 mM benzamidine, 1 μM aprotinin A, 1 μM pepstatin, 1 μM leupeptin, 1 mM PMSF, pH 7.4). Supernatants were loaded on a SDS-PAGE gel and transferred to PVDF membranes. After blocking for 1 h at room temperature with PBS buffer containing 6% non-fat milk powder, membranes were incubated with mouse anti-p53 primary antibody (2 μg/mL, DO-7, DAKO) at 4°C overnight and were subsequently incubated with the secondary HRP-IgG antibody at room temperature for 1 h.

### Detection of apoptosis through FCM

After collection, the cells were washed with ice-cold PBS buffer twice. Cell concentration was adjusted to 2 × 10^6^ cells/mL. Subsequently, Annexin-V/FITC solution, PI solution, and HEPES buffer were mixed together (1:50). Moreover, 100 μL Annexin-V-FITC/PI solution with 10^6^ cells was added into a EP tube and incubated for 15 min at room temperature. Finally, 1 mL HEPES buffer was added into the reaction tube, and the apoptosis index of the cells was detected using FCM within 1 h.

### Effect of p53 RNAi on ZR-75-30 and MDA-MB-231 apoptosis

To study the effect of the p53 RNAi on the high apoptosis indices of ZR-75-30 and MDA-MB-231, we constructed the pAd-p53-RNAi. Then, 1 × 10^5^ BC cells were inoculated into a 12-well plate. During transfection, the plasmid mix was added gradually into each well that contains tumor cells and media. The solution was mixed well by gradual shaking. After 48 h transfection, cells were collected via FCM method to detect apoptosis.

### Statistical analysis methods

All data were expressed as median values ± standard error (SE). The statistical significance of the differences in the measured values among groups was evaluated using one-way ANOVA. For individual comparisons, an independent unpaired t-test was used. All analyses were performed using the SPSS software package (SPSS, Chicago, IL, USA). Values were considered to have statistically significant difference if *p* < 0.01 or *p* < 0.05.

### Consent

Written informed consent was obtained from the patient for the publication of this report and any accompanying images.

## Competing interests

The authors declare that they have no co interests.

## Authors’ contributions

CSW and CFG were responsible for design of the study, performed the statistical analysis and manuscript preparation. XXL, JY and YPC participated in the discussion for histological characteristics, RT-PCR, transfection and manuscript preparation. PW cultured the cell lines and carried out the FCM detection. All authors read and approved the final manuscript.
